# Loss of LpqM proteins in *Mycobacterium abscessus* is associated with impaired intramacrophage survival

**DOI:** 10.1128/spectrum.03837-23

**Published:** 2024-04-15

**Authors:** Yves-Marie Boudehen, Wassim Daher, Françoise Roquet-Baneres, Laurent Kremer

**Affiliations:** 1 Centre National de la Recherche Scientifique UMR 9004, Institut de Recherche en Infectiologie de Montpellier (IRIM), Université de Montpellier, Montpellier, France; 2 INSERM, IRIM, Montpellier, France; Rutgers New Jersey Medical School, Newark, New Jersey, USA

**Keywords:** *Mycobacterium abscessus*, LpqM, lipoprotein, macrophage, infection, morphotype

## Abstract

*Mycobacterium abscessus,* an emerging pathogen responsible for severe pulmonary infections in cystic fibrosis patients, displays either a smooth (S) or a rough (R) morphotype. Infections with *M. abscessus* R are associated with increased pathogenicity in animal models and humans. While the S-to-R transition correlating with reduced glycopeptidolipid (GPL) production is well-documented, the recent screening of a transposon library revealed additional gene candidates located outside of the *GPL* locus involved in this transition. These genes include *MAB_1470c*, encoding the putative lipoprotein peptidase LpqM. However, experimental confirmation of the implication of this gene in the morphotype switch is lacking. Herein, we re-examined the role of *MAB_1470c*, and its homolog *MAB_1466c*, in colonial morphotype changes by generating unmarked deletion mutants in *M. abscessus* S. Our results indicate that the morphotype of these mutants stayed smooth in different media. Unexpectedly, the intracellular growth of Δ*MAB_1470c* and Δ*MAB_1466c* in THP-1 macrophages was significantly reduced as compared to the parental S strain, and these defects were rescued upon complementation with their corresponding genes. Strikingly, the intracellular survival defect was further exacerbated in a mutant lacking both *MAB_1470c* and *MAB_1466c* genes. This implies that, despite their primary sequence relatedness, the two proteins are not functionally redundant. Collectively, this suggests that these two LpqM-related lipoproteins are unlikely to be involved in the S-to-R transition but are key players for intramacrophage survival of *M. abscessus*.

**IMPORTANCE:**

*Mycobacterium abscessus* causes persistent infections in patients with underlying pulmonary diseases, resulting in progressive lung function deterioration. The rough (R) morphotype is well-established as associated with chronic and more aggressive infections in patients. In this study, we individually and simultaneously deleted the *MAB_1470c* and *MAB_1466c* genes in *M. abscessus* S, without observing changes in colony morphotypes. However, these mutants exhibited a severe impairment in their ability to survive within human macrophages, highlighting the critical role of these two lipoproteins in *M. abscessus* virulence.

## OBSERVATION

The ability of *M. abscessus* to alter its morphology and surface properties is a significant factor in its pathogenesis (1, 2). The production of glycopeptidolipids (GPL) determines the smooth (S) morphotype, while defects in GPL synthesis and/or transport are correlated with the emergence of a rough (R) variant, characterized by increased virulence (3, 4). Epidemiological studies have emphasized the greater impact of the *M. abscessus* R strain in severe lung infections and chronic colonization of airways in patients (5–7). The R variant forms “cords” *in vitro* and also in the zebrafish infection model (8, 9) and cords represent an immune evasion mechanism, as these structures are too large to be engulfed by professional phagocytes (9). Notably, the lack of GPL in the R variant is compensated by increased production of cell surface-associated lipoproteins (10), which serve as TLR2 agonists, explaining the intense pro-inflammatory response associated with *M. abscessus* R infections. The molecular events leading to the S-to-R transition have been extensively studied (4), with a particular focus on the presence of small insertions/deletions and single-nucleotide polymorphisms within the cluster of genes responsible for GPL biosynthesis and transport. These genetic alterations provide a satisfactory explanation for the irreversibility of the S-to-R phenotypic transition (4, 11). Single-nucleotide deletions were identified in *mmpL4b*, encoding a GPL transporter, and nucleotide insertions in *mps1*, responsible for encoding a non-ribosomal peptide synthase required for the synthesis of the tripeptide-aminoalcohol moiety in R variants as compared to their corresponding S counterparts. These genetic lesions represent some of the most frequently observed morphological alterations (4, 11). Studies in *M. abscessus* S revealed also that deletion of *gtf1* or *gtf2*, which encode the glycosyltransferases transferring the 6-deoxytalose and the first rhamnose onto the GPL peptide backbone, respectively, resulted in an S-to-R conversion. This transition was associated with an increased capacity to produce cords and abscesses and correlated with enhanced virulence in zebrafish (12). By screening a saturated Himar-1 transposon-mutant library in *M. abscessus* S, Foreman et al. identified 89 unique insertions in coding regions or noncoding regions associated with the shift in morphology from S to R (13). While most insertions are found within genes of the *GPL* cluster, as expected, some were also identified outside of this locus. Among these, *MAB_1470c* has been proposed as a potential candidate involved in the S-to-R colony morphology transition (13). This gene is orthologous to the lipoprotein metalloproteinase *lpqM* gene, required for conjugal DNA transfer in *Mycobacterium smegmatis* (14). These observations prompted us to re-examine the role of *MAB_1470c* (and its homolog *MAB_1466c*) in S-to-R transition through the production of genetically defined unmarked deletion mutants and to elucidate their potential implications in intracellular survival.

**Fig 1:**
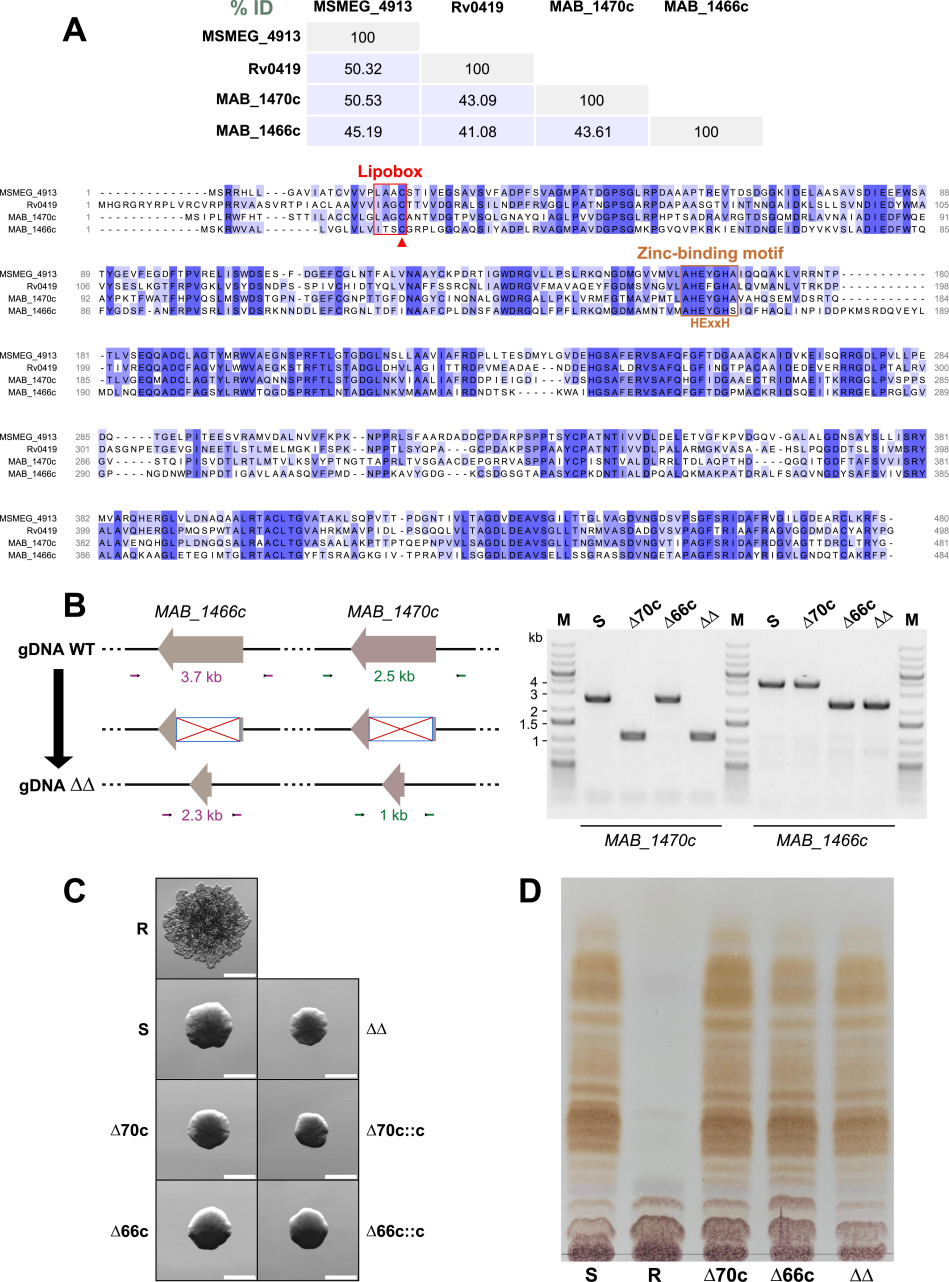
Deletion of *lpqM*-like genes does not alter *M. abscessus* colonial morphology. (**A**) The upper panel depicts the percentage of identity, while the lower panel displays a multiple sequence alignment of the LpqM-like proteins MAB_1470c and MAB_1466c from *M. abscessus*, MSMEG_4913 from *M. smegmatis,* and Rv0419 from *M. tuberculosis*. Sequences were retrieved from Mycobrowser and aligned using Clustal Omega and Jalview software. Conserved residues are highlighted in blue, and the characteristic lipobox and zinc-binding motif (HExxH) are indicated by red and brown boxes, respectively. The red arrowhead points to the conserved cysteine residue in the lipobox. (**B**) The left panel presents a schematic representation of the inactivation of *lpqM*-like genes in *M. abscessus*. The right panel shows an agarose gel image of PCR amplicons using gDNA from the parental *M. abscessus* S strain (S), Δ*MAB_1470c* (Δ70c), Δ*MAB_1466c* (Δ66c), and Δ*MAB_1470c*/Δ*MAB_1466c* (ΔΔ) mutants, along with primers producing a 2.5 kb fragment in the parental strain and a 1 kb fragment in Δ70c or a 3.7 kb fragment in the parental strain and a 2.3 kb fragment in Δ66c. Proper gene deletion was confirmed by DNA sequencing. M, molecular size marker. (**C**) Colony morphology of the parental S strain (S), the corresponding R variant (R), Δ70c and Δ66c, their respective complemented strains (Δ70c::c and Δ66c::c) and the double mutant Δ*MAB_1470c*/Δ*MAB_1466c* (ΔΔ). Pictures were captured after 4 days of incubation at 37°C on LB agar. Scale bar, 0.5 mm. (**D**) Thin-layer chromatography analysis of the GPL-containing lipid fraction of the parental strain (S), Δ70c, Δ66c, and ΔΔ mutants. The *M. abscessus* R variant, lacking GPL, is included as a control.

BLAST analyses showed that MAB_1470c shares 50% of protein identity with MSMEG_4913, a previously characterized lipoprotein metalloproteinase, known as LpqM in *M. smegmatis* (14) and a 43% identity with Rv0419 from *M. tuberculosis* (Fig. 1A). Bioinformatic searches also identified *MAB_1466c* as a homolog of *MAB_1470c* in *M. abscessus*. Multiple sequence alignments emphasized the presence of a typical lipobox housing a conserved cysteine residue at the N-terminus, which serves as the canonical site for lipid modification (15), as well as a zinc-binding motif characterized by the HExxH motif required for protease activity (Fig. 1A). While no information is currently available regarding the role of LpqM-like proteins in *M. abscessus* physiology and infection, we generated an unmarked deletion mutant of *MAB_1470c* (Δ70c) in the S variant of *M. abscessus* CIP104536^T^ (Fig. 1B) (16) using plasmids and primers listed in Tables S1 and S2, respectively. Evaluation of the morphotype of Δ70c indicated that the mutant maintained a smooth appearance on LB agar plates (Fig. 1C), a phenotype also confirmed on Middlebrook 7H10 and tryptic soy agar (data not shown). Complementation with the wild-type *MAB_1470* gene did not alter the S morphotype either. This suggests that the deletion of *MAB_1470c* is unlikely to contribute to the S-to-R transition in contrast to a previous study where a transposon insertion into *MAB_1470c* was associated with an R appearance (13). However, it is worth noting that neither images for assessing the roughness of the transposon mutant nor complementation studies demonstrating a reversion of the morphotype were provided in that study (13). Given the homology and proximity between *MAB_1466c* and *MAB_1470c* in the genome, which could potentially explain overlapping functions that rescue the phenotype in Δ70c, we also deleted *MAB_1466c* (Δ66c) (Tables S1 and S2). Similar to Δ70c, the Δ66c mutant displayed a smooth appearance on LB agar (Fig. 1C), Middlebrook 7H10, and tryptic soy agar (data not shown). Moreover, a double-mutant lacking both genes (designated ΔΔ) displayed a morphotype similar to the parental S strain (Fig. 1C). Since rough *M. abscessus* strains are typically characterized by defects in the synthesis and/or transport of GPL (4, 17), we analyzed the GPL profile of the single and double *lpqM*-like mutants. Thin-layer chromatography patterns clearly showed that all three mutants produced GPL at levels comparable to the parental S strain (Fig. 1D). Altogether, this suggests that the inactivation of *MAB_1470c* and/or *MAB_1466c* has no discernable impact on colonial morphology and that these genes are unlikely candidates involved in the S-to-R transition.

**Fig 2:**
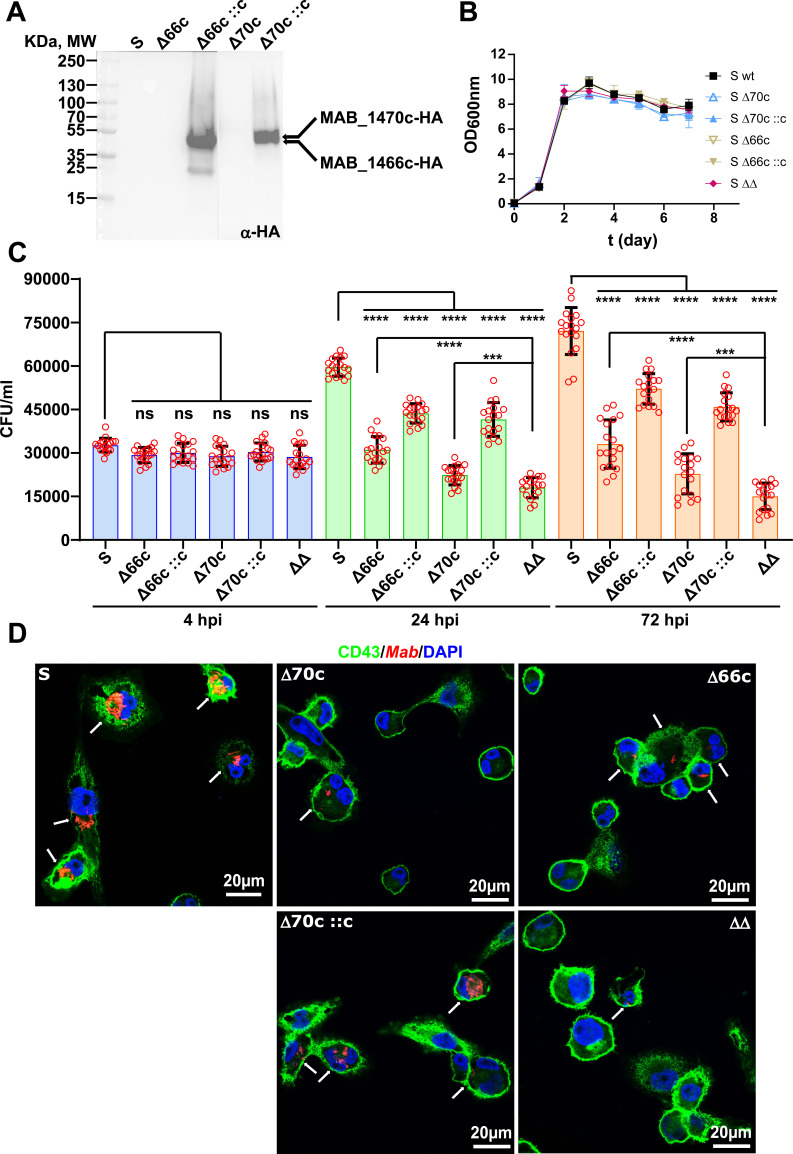
MAB_1470c and MAB_1466c are crucial for intracellular survival of *M. abscessus*. (A) Western blot showing the expression of MAB_1466c-HA and MAB_1470c-HA proteins in Δ66c::c and Δ70c::c, carrying the pMV361-*MAB_1466c*-HA and pMV361-*MAB_1470c*-HA, respectively. Membranes were probed with anti-HA antibodies. (B) Growth curves of the different strains in Middlebrook 7H9 medium at 37°C under shaking. Data are expressed as mean values ± SD from three independent experiments. (C) Macrophages were infected with different *M. abscessus* strains expressing tdTomato (MOI of 2:1) and CFU were determined at 4, 24, and 72 hpi. Data are presented as mean values ± SD from three independent experiments. One-tailed Tukey’s multiple comparisons test: ns, non-significant; ***P < 0.001; ****P < 0.0001. (D) Immunofluorescent fields were taken after 72 hpi at a 40× magnification using a confocal microscope, showing the nuclei (blue) and the periphery of the macrophages (green) infected with various *M. abscessus* strains (red), as indicated. White arrows point to mycobacteria-infected cells.

Δ70c and Δ66c were transformed with the integrative pMV361-*MAB_1470c*-HA and pMV361-*MAB_1466c*-HA, respectively. In these plasmids, *MAB_1470c* and *MAB_1466c* were placed under the control of the *hsp60* promoter and fused with an HA-tag at the 3′-end (Table S1). Immunoblotting of crude lysates, probed with anti-HA antibodies, confirmed the production of MAB_1470c and MAB_1466c in the corresponding complemented strains (designated Δ70c::c and Δ66c::c) (Fig. 2A). Deletion of the *lpqM*-like genes had no discernable impact on the replication rate of *M. abscessus* in planktonic culture in 7H9 (Fig. 2B) or in M63 medium (Fig. S1), nor did it alter sliding motility on 7H9-0.3% agar (Fig. S2). Given the typical association of lipoproteins with the cell wall, we assessed the drug susceptibility profile of the different strains against a broad panel of clinically used drugs. Table S3 shows that all strains exhibited comparable drug susceptibility to the parental S strain.

We next investigated the potential impact of these deletions on adhesion and invasion of *M. abscessus* by human THP-1 macrophages, as previously described (12, 18). Cells were infected with *M. abscessus* S, Δ70c, and Δ66c along with their complemented strains and the double mutant, all expressing tdTomato (9), for 4 h at a multiplicity of infection (MOI) of 2:1. Following infection, macrophages were treated with 250 µg/mL amikacin for 2 h, and amikacin was maintained at 50 µg/mL to prevent extracellular bacterial growth (12, 18). At 4, 24, and 72 h post-infection (hpi), cells were lysed and plated to determine the intracellular bacterial burden. At 4 hpi, the invasion rate of all strains was similar (Fig. 2C). However, at 24 hpi, the growth rate of Δ66c and Δ70c was reduced by ~48% and 62%, respectively, compared to the parental S strain, and this effect was partially rescued upon complementation (Fig. 2C). The reduced intracellular growth defect of the single mutants was even more pronounced at 72 hpi. Importantly, this effect was further exacerbated in the double mutant, with ~80% reduction in CFUs compared to the control strain (Fig. 2C). This suggests that *MAB_1470c* and *MAB_1466c* cannot complement each other in *M. abscessus*. At 72 hpi, macrophages were stained with DAPI and anti-CD43 antibodies and imaged using confocal microscopy (12). These observations emphasize a more pronounced reduction in the number of THP-1 cells infected with the single mutants and a near-complete absence of cells infected with the double mutant, compared to infection with the parental S strain (Fig. 2D), in agreement with the CFU results (Fig. 2C).

In conclusion, the attenuated phenotype of Δ*MAB_1470c* and Δ*MAB_1466c* in macrophages underscores the importance of these putative lipoproteins in *M. abscessus* virulence, consistent with the attenuation of *Mycobacterium tuberculosis* mutants lacking other lipoproteins (15, 19). Future studies are required to identify the substrate(s) of these LpqM proteases. The pulldown purification of affinity-tagged LpqM, incubated with *M. abscessus* lysates and/or culture filtrates, followed by mass-spectrometry analysis, would help in identifying LpqM substrates or protein partners, as previously reported for the lipoprotein LpqN in *M. tuberculosis* (20). Subsequent characterization of the substrate mutants will provide new insights into the role of LpqM and other lipoproteins in mycobacterial cell wall biology and the pathogenesis of *M. abscessus*.

## Data Availability

All data generated in this study are available upon request.

## SUPPLEMENTAL MATERIAL

Supplemental material spectrum.03837-23-s0001.pdf Table S1 to S3; Fig. S1 and S2.

ASM does not own the copyrights to Supplemental Material that may be linked to, or accessed through, an article. The authors have granted ASM a non-exclusive, world-wide license to publish the Supplemental Material files. Please contact the corresponding author directly for reuse.
