# Supplementary material for: Loss of LpqM proteins in Mycobacterium abscessus is associated with impaired intramacrophage survival
Source: Microbiol Spectr. 2024 Apr 15;12(5):e03837-23. doi: 10.1128/spectrum.03837-23 (PMC11064476; doi:10.1128/spectrum.03837-23)
Supplement: Supplemental material — Table S1 to S3; Fig. S1 and S2. [file spectrum.03837-23-s0001.pdf]

Table S1. Design of the constructs and plasmids used in this study.

Plasmid	Parent vector/ Selection	Cloning technique	Design of the constructs	Reference
pUX1-*katG*	Hyg^R, Kan^R		For doing unmarked deletions in *M. abscessus*	(1)
pUX1-*katG-atf1*	Kan^R		pUX1-*katG* containing *atf1*	(2)
pMV306	Kan^R		Integrative vector	(3)
pMV361-*atf1*,HA	pMV306 Kan^R		pMV306 containing *atf1*,HA under the control of the *hsp60* promotor.	(2)
[a]pUX1-*katG-MAB_1470c*	pUX1-*katG- atf1* Kan^R	In-Fusion reaction	The pUX1-*katG* backbone was PCR-amplified with pUX backbone Left / pUX backbone Right primers. Up- and down-stream PCR fragments of *MAB_1470c* were amplified using *M. abscessus* genomic DNA as the template with mab_1470c-ko up (F) / mab_1470c-ko up (R) and mab_1470c-ko dw (F) / mab_1470c-ko dw (R) primers. Then, the pUX1-*katG* backbone and the two amplified PCR fragments were ligated through In-Fusion reaction.	This work
[a]pUX1-*katG-MAB_1466c*	pUX1-*katG-atf1* Kan^R	In-Fusion reaction	The pUX1-*katG* backbone was PCR-amplified with pUX backbone Left / pUX backbone Right primers. Up- and down-stream PCR fragments of *MAB_1466c* were amplified using *M. abscessus* genomic DNA as the template with mab_1466c-ko up (F) / mab_1466c-ko up (R) and mab_1466c-ko dw (F) / mab_1466c-ko dw (R) primers. Then, the pUX1-*katG* backbone and the two amplified PCR fragments were ligated through In-Fusion reaction.	This work
pMV361-*MAB-1470c,HA*	pMV361-*atf1*,HA Kan^R	In-Fusion cloning	The pMV361-*kan,HA* backbone was amplified using pMV361-*kan-atf1,HA* as a template and the primers pMV361-*hsp60* (Left) and pMV361-*hsp60*,HA (Right). *MAB_1470c* was amplified using *M. abscessus* genomic DNA as the template, with mab_1470c com-inf (F2) and mab_1470c,HA com-inf (R) primers. Both fragments were purified and ligated through In-Fusion reaction.	This work
pMV361-*MAB_1466c,HA*	pMV361-*atf1*,HA Kan^R	In-Fusion cloning	The pMV361-*kan,HA* backbone was amplified using pMV361-*kan-MAB_atf1,HA* as the template, along with the primers pMV361-*hsp60* (Left) and pMV361-*hsp60*,HA (Right). *MAB_1466c* was PCR-amplified using *M. abscessus* genomic DNA as the template and the mab_1466c com-inf (F2) and mab_1466c com-inf (R) primers. Both fragments were purified and ligated through In-Fusion reaction.	This work
pTEC27	Hyg^R		For expression of tdTomato	(4)

[a]pUX1-*katG-MAB_1470c* and pUX1-*katG-MAB_1466c* were engineered to delete 98.9% and 99.2% of the open reading frames of *MAB_1470c* and *MAB_1466c*, respectively.

Table S2: Primers used in this study.

Name	Sequence 5' → 3'
pUX backbone Left	GCTAGCACACCAGACAAGTTGG
pUX backbone Right	TTAATTAAGTTAACTAGCGTACGATCG
mab_1470c-ko up (F)	TAGTTAACTTAATTAAgctggcattggcctgttggg
mab_1470c-ko up (R)	gatcgacatggccgtctctctg
mab_1470c-ko dw (F)	gacggccatgtcgatctacgggtgatgatcgcgccg
mab_1470c-ko dw (R)	TGTCTGGTGTGCTAGCtctcgatgagcagcatccgg
mab_1466c-ko up (F)	TAGTTAACTTAATTAActggactccgaacatccacgg
mab_1466c-ko up (R)	tttggacatcaagccacccctttg
mab_1466c-ko dw (F)	tggcttgatgtccaaaccctagctcctaaccggcacg
mab_146cc-ko dw (R)	TGTCTGGTGTGCTAGCacaaggctggcaagttgccg
mab_1470c com-inf (F2)	GGCCAAGACAATTGCCcatgtcgatcccgctcaggtg
mab_1470c,HA com-inf (R)	GAACATCGTATGGGTAcccgtagcgggtgaggcag
mab_1466c com-inf (F2)	GGCCAAGACAATTGCCccatgtccaaacgctgggtagca
mab_1466c com-inf (R)	GAACATCGTATGGGTAggggaagcgcttggcgcag
pMV361-hsp60 (Left)	GGCAATTGTCTTGGCCATTG
pMV361-hsp60 HA (Right)	TACCCATACGATGTTCCAGATTACG

Table S3. MIC of drugs against *M. abscessus lpqM* mutants.

Antibiotic		MIC (µg/ml) (phenotype)					
	Range (µg/ml)	S	Δ*66*	Δ*66c::c*	Δ*70c*	Δ*70c::c*	ΔΔ
Trimethoprim/ Sulfamethoxazole	0.25/4.75-8/152	>8/152 (R)	>8/152 (R)	>8/152 (R)	>8/152 (R)	>8/152 (R)	>8/152 (R)
Linezolid	1-32	>32 (R)	32 (R)	>32 (R)	>32 (R)	32 (R)	32 (R)
Ciprofloxacin	0.12-4	>4 (R)	>4 (R)	>4 (R)	>4 (R)	>4 (R)	>4 (R)
Imipenem	2-64	64 (R)	64 (R)	64 (R)	64 (R)	>64 (R)	64 (R)
Moxifloxacin	0.25-8	>8 (R)	>8 (R)	>8 (R)	>8 (R)	>8 (R)	>8 (R)
Cefepime	1-32	>32(R)	>32 (R)	>32 (R)	>32 (R)	>32 (R)	>32 (R)
Cefoxitin	4-128	64 (I)	64 (I)	64 (I)	64 (I)	128 (R)	64 (I)
Amoxicillin/ Clavulanic acid	2/1-64/32	>64/32 (R)	>64/32 (R)	>64/32 (R)	>64/32 (R)	>64/32 (R)	>64/32 (R)
Amikacin	1-64	32(I)	32 (I)	32 (I)	64(R)	32 (I)	32 (I)
Ceftriaxone	4-64	>64 (R)	>64 (R)	>64 (R)	>64 (R)	>64 (R)	>64 (R)
Doxycycline	0.12-16	>16 (R)	>16 (R)	>16 (R)	>16 (R)	>16 (R)	>16 (R)
Minocycline	1-8	>8 (R)	>8 (R)	>8 (R)	>8 (R)	>8 (R)	>8 (R)
Tigecycline	0.015-4	2 (S)	1 (S)	2 (S)	1 (S)	2 (S)	1 (S)
Tobramycin	1-16	>16 (R)	>16 (R)	>16 (R)	>16 (R)	>16 (R)	>16 (R)
Clarithromycin (4 days)	0.06-16	>16 (R)	8 (R)	>16 (R)	16 (R)	>16 (R)	16 (R)

The phenotype was attributed according to the breakpoints for rapidly-growing mycobacteria (5). S: susceptible. R: resistant. I: intermediate.

REFERENCES

1. Richard M, Gutiérrez AV, Viljoen A, Rodriguez-Rincon D, Roquet-Baneres F, Blaise M, Everall I, Parkhill J, Floto RA, Kremer L. 2019. Mutations in the MAB_2299c TetR Regulator Confer Cross-Resistance to Clofazimine and Bedaquiline in *Mycobacterium abscessus*. Antimicrob Agents Chemother 63.
2. Illouz M, Leclercq L-D, Dessenne C, Hatfull G, Daher W, Kremer L, Guérardel Y. 2023. Multiple *Mycobacterium abscessus* O-acetyltransferases influence glycopeptidolipid structure and colony morphotype. J Biol Chem 299:104979.
3. Stover CK, de la Cruz VF, Fuerst TR, Burlein JE, Benson LA, Bennett LT, Bansal GP, Young JF, Lee MH, Hatfull GF. 1991. New use of BCG for recombinant vaccines. Nature 351:456–460.
4. Takaki K, Davis JM, Winglee K, Ramakrishnan L. 2013. Evaluation of the pathogenesis and treatment of *Mycobacterium marinum* infection in zebrafish. Nat Protoc 8:1114–1124.
5. Hatakeyama S, Ohama Y, Okazaki M, Nukui Y, Moriya K, Antimicrobial susceptibility testing of rapidly growing mycobacteria isolated in Japan. 2017. BMC Infect Dis 17:197.

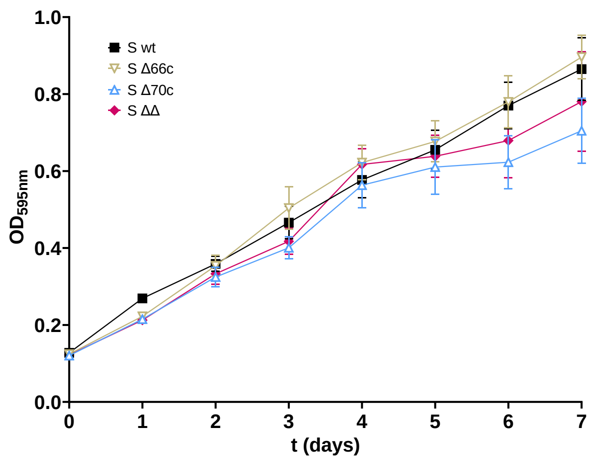

Figure S1. Growth curves of *M. abscessus* Δ*lpqM* strains in M63 Medium. A volume of 200 microliters of each strain at an OD of 0.1 was dispensed into individual wells of a treated flat-bottom 96-well plate. The plates underwent static incubation at 37°C for 7 days with measurements recorded daily using a spectrophotometric multimode microplate reader (Tecan Spark 10M; Tecan Group Ltd., Switzerland). This experimental setup consisted of 4 technical replicates and three biological replicates per strain.

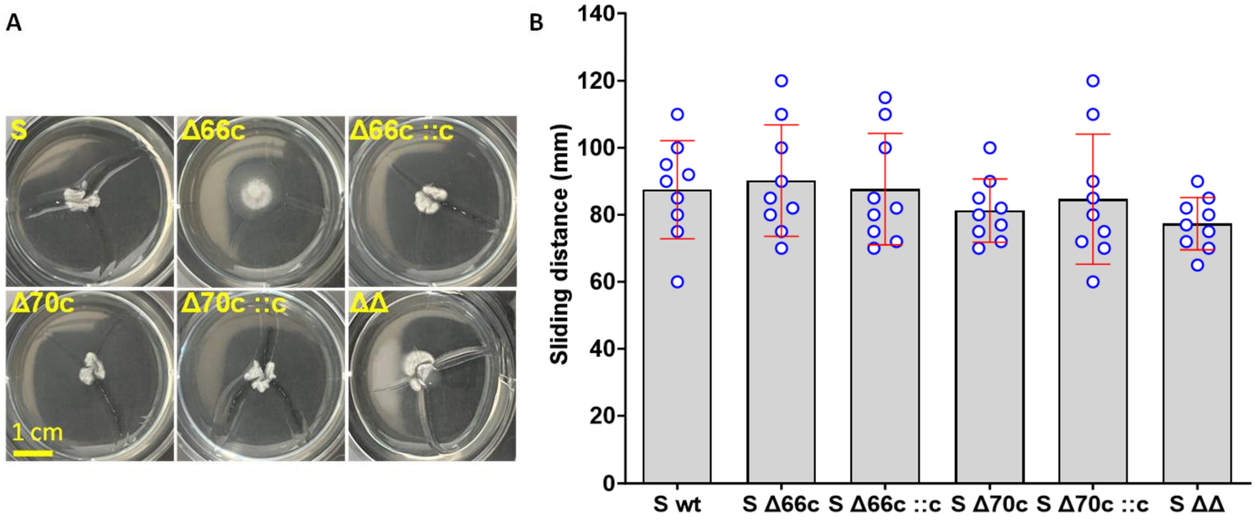

Figure S2. Surface sliding capabilities of *M. abscessus* strains. (A) The assessment involved observing the sliding abilities of *M. abscessus* wild-type S, Δ*lpqM* mutants and complemented strains on the surfaces of 7H9-0.3% agar medium. **(B)** The sliding distances (in mm), depicted as the means of three independent experiments, were plotted for all strains. No discernable reduction in the sliding fitness of the strains was observed.
